# Perceived Usefulness of Digital Assistance Systems Among Psychology Students and Psychotherapists: Predictors, Barriers and the Impact of a Brief Video Demonstration

**DOI:** 10.1002/ijop.70268

**Published:** 2026-09-24

**Authors:** Michelle Oberthaler, Bonnie Röhrig, Frank Petrak, Stephan Goerigk

**Affiliations:** ^1^ Charlotte Fresenius Hochschule Wiesbaden/München Germany; ^2^ Zentrum für Psychotherapy MVZ GmbH Wiesbaden Germany; ^3^ Department of Psychosomatic Medicine and Psychotherapy University Clinic Bochum, Ruhr‐University Bochum Bochum Germany; ^4^ iptas INNOVATIONS GmbH Wiesbaden Germany

**Keywords:** artificial intelligence, digital assistance systems, perceived usefulness, psychotherapy, technology acceptance, work‐related stress

## Abstract

Digital assistance systems based on artificial intelligence are increasingly discussed as tools to address growing demands in mental health care. However, their acceptance among future and practising psychotherapists remains insufficiently understood. In a pre‐post online survey, *N* = 317 participants (predominantly psychology students, with smaller groups of trainee psychotherapists and licensed psychotherapists) completed questionnaires assessing perceived usefulness (PU), work‐related stress, technological affinity, professional self‐efficacy, data protection concerns and prior knowledge. Between assessments, participants watched a video demonstrating a digital assistance system. An open‐ended item captured perceived barriers. PU increased significantly after the video (*p* < 0.001, *d* = 0.29), with subgroup differences across professional groups. Effects were largest among trainee psychotherapists (*d* = 0.59) and negligible among licensed psychotherapists (*d* = 0.06). Work‐related stress was positively associated with PU, whereas age was negatively associated. Technological affinity, prior knowledge, professional self‐efficacy and data protection concerns showed no significant associations with PU. Open‐ended responses highlighted concerns regarding data protection, usability, time demands and professional autonomy. Brief, targeted demonstrations may increase PU of digital assistance systems among psychology students, although effects appear minimal in licensed psychotherapists. The mechanisms underlying the video effect and the positive association between stress and PU warrant further investigation.

## 1 Introduction

### 1.1 Digitalisation in Mental Health Care

Digitalisation is transforming economic and social systems worldwide, with particularly profound implications for healthcare (Delicat et al. 2024). In mental health care, digital tools such as mobile health applications, wearable sensor technologies and artificial intelligence are increasingly used to support therapeutic practice (Hirjak et al. 2022). This rapid innovation coincides with a global mental health crisis: more than one billion people were living with a mental disorder in 2021, representing approximately 14% of the global population (World Health Organization 2025). The lingering effects of the COVID‐19 pandemic have further intensified demand for services that many health systems are unable to meet (Lebedyn et al. 2024). Even in high‐income countries, only one third of individuals with major depressive disorder receive appropriate treatment (World Health Organization 2025).

Against this backdrop, digital technologies are increasingly regarded as essential complements to conventional therapy. They may address critical gaps in mental health care by broadening access, reducing professional workload and streamlining routine tasks (Hirjak et al. 2022).

### 1.2 Digital Assistance Systems in Psychotherapy

Digital technologies offer new pathways for psychotherapy, ranging from fully digital interventions to blended formats that combine technology with face‐to‐face therapy (Fuhr et al. 2024). Although self‐guided applications often face challenges such as low engagement and limited feedback, their integration into clinical practice has shown benefits for motivation, processing of distressing content and therapeutic effectiveness (Gumz and Boettcher 2021). These findings have been reported across different health systems, highlighting the global relevance of digital tools in mental health care (Beg and Verma 2024; Mohtar et al. 2025).

Within this spectrum, digital assistance systems are therapist‐directed support tools that augment, but do not replace, psychotherapy. Their core functions include diagnostic support, therapy planning, automated documentation and case management (Steidle et al. 2024). By incorporating artificial intelligence, they can process complex datasets efficiently, thereby reducing administrative workload and potentially improving treatment quality (LeCun et al. 2015; AlQudah et al. 2021).

Nevertheless, integration faces persistent barriers, including the absence of standardised data‐sharing frameworks, institutional reluctance to invest and concerns that digital tools may increase rather than decrease workload (Berardi et al. 2024; Hirjak et al. 2022). At the same time, enabling factors include person‐centred design, provider training, interprofessional collaboration and supportive policy reforms (Berardi et al. 2024).

### 1.3 Technology Acceptance: Theoretical Framework

The Technology Acceptance Model (TAM; Davis 1989) is the most widely applied framework for understanding individual‐level technology adoption. TAM posits that perceived usefulness (PU) and perceived ease of use (PEOU) are the primary determinants of behavioural intention to use a technology and, ultimately, of actual use. Extensions of TAM (TAM2, TAM3) and the Unified Theory of Acceptance and Use of Technology (UTAUT; Venkatesh et al. 2003) incorporate additional social, psychological and demographic influences. Empirical studies consistently confirm the predictive role of PU in health care contexts (AlQudah et al. 2021; Nazari‐Shirkouhi et al. 2023).

The present study employs TAM as a theoretical orientation rather than as a fully specified structural model. Specifically, PU serves as the primary outcome measure, consistent with its role as a central predictor of technology adoption within TAM (Venkatesh and Davis 2000). The study does not assess actual usage behaviour or intention to use. Because the system under investigation is not yet implemented in participants' routine clinical or educational environments, it is best understood as an early pre‐adoption evaluation rather than technology acceptance in the full behavioural sense. PU was therefore used as a theoretically relevant, but intentionally narrower, indicator of perceived value. Factors impeding adoption in this context include limited transparency, unclear responsibilities and mistrust toward commercial providers (Koch et al. 2020).

### 1.4 Predictors of PU

Building on the TAM framework, the present study investigates several individual‐level predictors theoretically relevant to psychotherapeutic practice. First, work‐related stress is examined. Prior research suggests that elevated occupational strain may reduce PEOU (Venkatesh et al. 2003) and foster rejection of digital tools (Kothgassner et al. 2012). Despite its relevance, stress has rarely been examined in technology acceptance research in psychotherapy contexts (Kulik et al. 2023). We therefore hypothesised that higher stress would be negatively associated with PU.

Second, technological affinity is examined as an individual predisposition that may facilitate acceptance. Third, professional self‐efficacy—confidence in managing professional challenges—was examined exploratorily as a potential correlate of PU and as a possible moderator of stress‐related effects, without a directional hypothesis. Fourth, data protection concerns are assessed given the sensitivity of clinical data; stronger privacy concerns were hypothesised to be negatively associated with PU. Finally, prior knowledge of digital applications is examined as a contextual covariate.

### 1.5 Research Questions

Does exposure to a brief video demonstration increase PU of a digital assistance system?

Are technological affinity, work‐related stress, professional self‐efficacy, data protection concerns and prior knowledge associated with PU?

Is age associated with PU?

What qualitative barriers do participants report regarding the use of digital assistance systems?

By addressing these questions across professional stages, the study aims to generate insights informing implementation strategies, therapist training and policy development.

## 2 Methods

### 2.1 Ethics

This study was conducted in accordance with the ethical principles of the 1964 Declaration of Helsinki and its later amendments. The study consisted of an anonymous, minimal‐risk online survey that included an informational video stimulus; it involved no clinical intervention and no collection of sensitive personal data beyond participants' professional background. Under applicable German legal and professional regulations, formal ethics committee approval was therefore not required. All participants were informed of the study's purpose and provided informed consent electronically prior to participation.

### 2.2 Research Design

This study employed a quantitative pre‐post online survey design. PU was assessed before and after a brief informational video stimulus. Sociodemographic, professional and psychological variables were collected to examine potential predictors and covariates. Data were collected between 25 May 2025 and 17 June 2025 using Unipark (EFS‐Survey), a platform compliant with European data protection regulations (GDPR).

### 2.3 Participants

A total of 317 participants were included in the final sample, exceeding the required sample size of 200 identified through a priori power analysis (*M*_age_ = 25.91, SD = 9.60; range: 18–69 years; 82.0% female). The sample was predominantly composed of psychology students (82.6%; *n* = 262), followed by licensed psychotherapists (11.7%; *n* = 37) and trainee psychotherapists (4.7%; *n* = 15). This uneven distribution limits the interpretability of subgroup‐specific inferential analyses (see Section 4.2).

**FIGURE 1:**
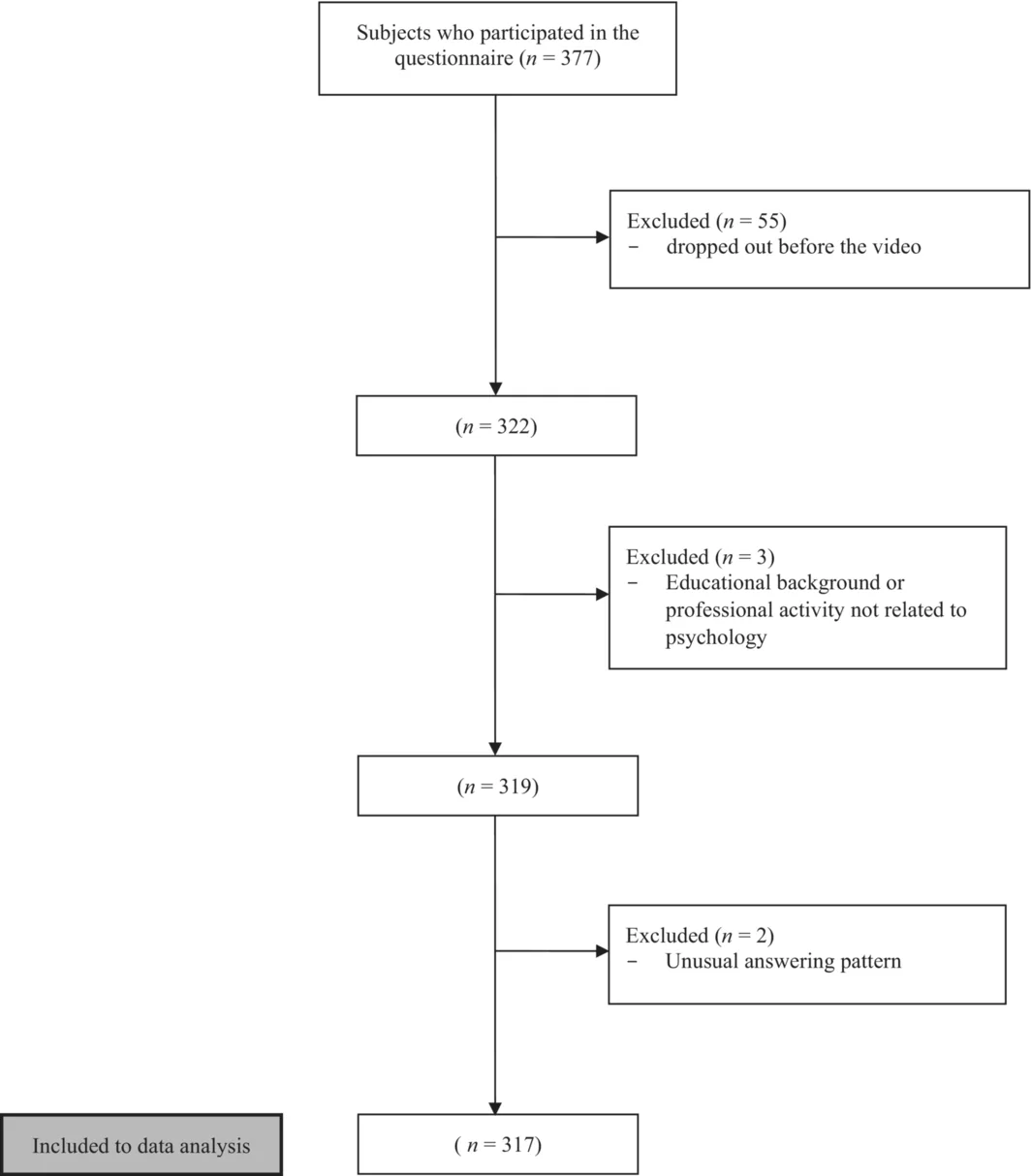
CONSORT flow diagram.

Inclusion criteria: licensed psychotherapists, trainee psychotherapists or psychology students actively enrolled during the data collection period, with access to a digital device. Exclusion criteria: no relevant academic or professional background in psychology, absence of informed consent, completion of fewer than two questionnaire sections, or dropout before the post‐video assessment. The exclusion process is illustrated in the CONSORT flow diagram (Figure 1).

### 2.4 Measures

#### 2.4.1 PU (Primary Outcome)

PU was assessed using four items adapted from Davis (1989) and Venkatesh and Davis (2000), selected to reflect the psychotherapeutic context. Example item: ‘The use of the shown digital assistance system could improve my work performance.’ Responses were recorded on a 5‐point Likert scale from 1 (*does not apply at all*) to 5 (*applies completely*). Items were administered before and after the video. Mean scores were calculated; higher scores indicate greater PU. The scale was administered in German. Scale adaptation from a forced‐choice to a 5‐point Likert format was chosen for consistency across the questionnaire; this limits direct comparability with normative data and is acknowledged as a limitation (Cronbach's *α* = 0.86 pre‐video; *α* = 0.88 post‐video).

#### 2.4.2 Digital Application Knowledge

Three self‐developed items, based on the Digital Health Literacy Instrument (DHLI; van der Vaart and Drossaert 2017), assessed prior knowledge of digital applications (e.g., ‘I am familiar with the most important digital tools for psychotherapeutic practice’). Responses were recorded on a 5‐point Likert scale. Mean scores were calculated (*α* = 0.89). Items were administered in German.

#### 2.4.3 Technological Affinity

Technological affinity was measured using the short Affinity for Technology Interaction Scale (ATI‐S; Karrer‐Gauß et al. 2024). The original 6‐point scale was adapted to a 5‐point Likert format (1 = *strongly disagree*, 5 = *strongly agree*) for consistency across the questionnaire. This adaptation limits direct comparability with normative data from the original validation study and is acknowledged as a limitation. Two items were reverse‐scored per instrument guidelines. Mean scores were calculated; higher values indicate stronger technological affinity (*α* = 0.76). Items were administered in German.

#### 2.4.4 Work‐Related Stress

Six items adapted from the Copenhagen Psychosocial Questionnaire (COPSOQ; Kristensen et al. 2005) assessed work‐related stress (e.g., ‘I feel that I have a lot to do’). Responses were recorded on a 5‐point scale from 1 (*never*) to 5 (*always*). Mean scores were calculated (*α* = 0.86). For descriptive and visualisation purposes only, stress was dichotomised using a median split; all primary analyses treated stress as a continuous variable.

#### 2.4.5 Professional Self‐Efficacy

Six items from the Professional Self‐Efficacy Expectation Scale (Schyns and von Collani 2014) assessed confidence in managing professional challenges. Responses were recorded on a 5‐point Likert scale (1 = *strongly disagree*, 5 = *strongly agree*). Mean scores were calculated (*α* = 0.83).

#### 2.4.6 Data Protection Concerns

Four items adapted from the Internet Users' Information Privacy Concerns scale (IUIPC; Malhotra et al. 2004) assessed privacy concerns. Responses were recorded on a 5‐point Likert scale. Mean scores were calculated (*α* = 0.89).

#### 2.4.7 Open‐Ended Item: Personal Barriers

Participants were asked: ‘In your opinion, are there any other personal obstacles or difficulties in using digital assistance systems?’ (German: ‘Gibt es aus Ihrer Sicht weitere persönliche Hindernisse oder Schwierigkeiten bei der Nutzung digitaler Assistenzsysteme?’). Responding was optional to minimise dropout. Responses were analysed by the first author using inductive thematic analysis (Braun and Clarke 2006). Consistent with a reflexive thematic analysis approach, coding was treated as an interpretative rather than a measurement procedure; intercoder reliability was therefore not assessed.

#### 2.4.8 Informational Video Stimulus

A self‐produced, 6‐min 56‐s video was embedded midway through the survey. The video defined digital assistance systems, described core functions (structured treatment planning, AI‐supported documentation, automated progress monitoring, evidence‐based intervention suggestions) and addressed both anticipated benefits and critical perspectives (data protection, therapist responsibility, risk of technological dependency). Viewing was voluntary; playback completion was recorded. Because reliable completion data were not available for all participants, video completion is addressed as a limitation rather than through a formal sensitivity analysis. The video depicted a specific system (iptas), which limits generalisation of the observed PU change to other systems.

#### 2.4.9 Demographics and Background Variables

Sociodemographic data collected included age, gender (female, male, diverse), educational and professional background, psychotherapeutic orientation (multiple‐choice) and prior experience with digital assistance systems in psychotherapy (yes/no with free‐text field).

### 2.5 Statistical Analyses

All analyses were conducted using R (version 4.5.1). Descriptive statistics and reliability estimates (Cronbach's *α*) were calculated for all scales; mean scores were used throughout. Differences in PU before and after the video were examined using paired‐sample *t*‐tests when normality assumptions were met (assessed via Shapiro–Wilk tests) and Wilcoxon signed‐rank tests otherwise; effect sizes are reported as Cohen's *d*. Spearman's *ρ* was used for associations between continuous predictors and PU. Multiple linear regression and hierarchical regression with centred interaction terms were used to examine predictor effects and moderation. Group comparisons employed independent *t*‐tests, Wilcoxon tests, or one‐way ANOVAs; Welch adjustments were applied when homogeneity of variance was violated. Effect sizes are reported as *η* ^2^ and Cohen's *d*. Model assumptions (normality, homoscedasticity, multicollinearity) were systematically checked; robust standard errors (HC3) and bootstrap confidence intervals were used as supplementary checks. The significance level was set at *α* = 0.05.

Given the exploratory nature of a substantial portion of the analyses, no correction for multiple testing was applied; results involving multiple comparisons should therefore be interpreted with particular caution. This decision is consistent with common practice in exploratory technology acceptance research (Jokisch et al. 2022).

Subgroup analyses examined potential moderation by clinical experience. For inferential analyses, participants were categorised into two groups: psychology students (*n* = 262) and practitioners (trainee and licensed psychotherapists combined; *n* = 52). This grouping was chosen due to the small and unequal sizes of the practitioner subgroups (*n* = 15 and *n* = 37, respectively), which preclude adequately powered separate analyses. For all descriptive statistics and figures, results are reported separately for all three groups.

## 3 Results

### 3.1 Sample Characteristics and Descriptive Statistics

The final sample comprised 317 participants (*M*_age_ = 25.91, SD = 9.60; range: 18–69 years; 82.0% female (*n* = 260), 17.7% male (*n* = 56) and 0.3% diverse (*n* = 1)). Sample characteristics and descriptive statistics for all psychological variables (means, standard deviations, medians, reliability estimates) are presented in Table 1. All values are based on mean scores.

**TABLE 1:** Sample details and group differences.

	Students (*N* = 262)	Licensed psychotherapists (*N* = 37)	Trainee psychotherapists (*N* = 15)	*η* ^2^	Total (*N* = 314)
Age	22.9 ± 5.18	44.9 ± 11.4	29.7 ± 6.96	0.56	25.8 ± 9.52
Female	221 (84.4%)	25 (67.6%)	11 (73.3%)		257 (81.8%)
Male	41 (15.6%)	12 (32.4%)	3 (20.0%)		56 (17.8%)
Diverse	0 (0.0%)	0 (0.0%)	1 (6.7%)		1 (0.3%)
Perceived usefulness (pre)	3.88 ± 0.62	3.32 ± 1.01	3.50 ± 0.57	0.07***	3.79 ± 0.70
Stress	3.10 ± 0.71	2.83 ± 0.58	3.34 ± 0.39	0.02*	3.08 ± 0.69
Perceived usefulness (post)	4.09 ± 0.78	3.38 ± 1.20	3.80 ± 0.85	0.07***	3.99 ± 0.87
Data protection concerns	3.10 ± 1.01	3.39 ± 0.85	3.20 ± 0.94	0.01	3.14 ± 0.99
Function	3.74 ± 0.57	3.48 ± 0.90	3.75 ± 0.60	0.02	3.71 ± 0.62
Affinity	2.80 ± 0.81	2.91 ± 1.01	3.23 ± 0.87	0.01	2.83 ± 0.84
Level of information	1.97 ± 0.81	2.76 ± 0.98	2.53 ± 0.96	0.10***	2.09 ± 0.88
Self‐efficacy	4.15 ± 0.51	4.31 ± 0.48	4.19 ± 0.56	0.01	4.17 ± 0.51

*Note:* Individuals who selected ‘other’ on the educational‐level item (*n* = 3) were excluded. Plus‐minus values are means ± standard deviations; scale values are mean scores. Age and the scales perceived usefulness before and after the video (TAM), stress (COPSOQ), data protection concerns (DPCS), function, affinity (ATI‐S), level of information (DHLI) and self‐efficacy were tested using one‐way ANOVAs; gender distribution was tested using a chi‐square test. The effect size is eta squared. Asterisks indicate the significance level of the corresponding one‐way ANOVA.

* *p* < 0.05.

** *p* < 0.01.

*** *p* < 0.001.

### 3.2 Impact of Video on PU (RQ1)

**FIGURE 2:**
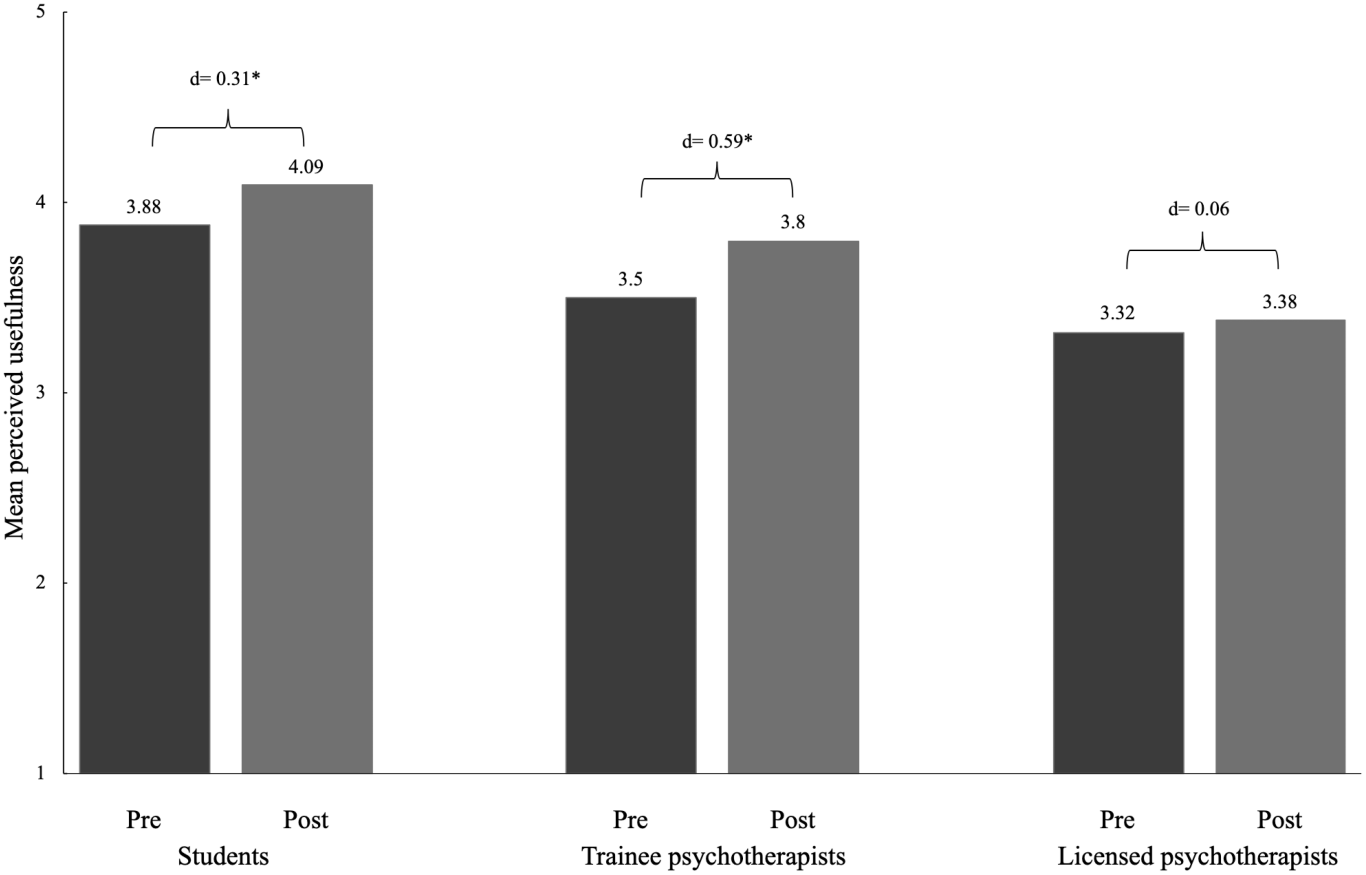
Pre‐post comparison of perceived usefulness by professional group. *N* = 314. Individuals who selected ‘other’ on the educational‐level item (*n* = 3) were not included. Within‐group pre–post changes were tested using the procedures described in Section 2.5. Effect sizes are reported as Cohen's *d*. *p < 0.05; **p < 0.01; ***p < 0.001.

PU increased significantly following the informational video stimulus (Wilcoxon signed‐rank test: *Z* = −5.76, *p* < 0.001, *r* = 0.32). As a sensitivity analysis, the paired *t*‐test yielded a consistent result, *t*(316) = −5.13, *p* < 0.001, *d* = 0.29, 95% CI [0.18, 0.40]. Descriptive results for all three groups are presented separately in Figure 2. The largest effect was observed among trainee psychotherapists (*d* = 0.59), followed by psychology students (*d* = 0.31). Licensed psychotherapists showed a negligible increase (*d* = 0.06), indicating that the video had a minimal effect in this group. The overall effect thus appears to be driven primarily by the student subsample.

### 3.3 Technological Affinity and Prior Knowledge (RQ2)

A weak positive association between technological affinity and PU was observed before the stimulus (*ρ* = 0.14, *p* = 0.013), which was no longer significant after the stimulus (*ρ* = 0.07, *p* = 0.204). Prior knowledge of digital applications, included as a contextual covariate, was not significantly associated with PU before (*ρ* = 0.032, *p* = 0.565) or after the video (*ρ* = −0.006, *p* = 0.914). Regression analyses corroborated these null findings (*B* = −0.002, SE = 0.020, *t*(315) = −0.08, *p* = 0.935, *R* ^2^ ≈ 0).

### 3.4 Work‐Related Stress (RQ2)

Contrary to the a priori hypothesis, work‐related stress showed a small but consistent positive association with PU, both before (*ρ* = 0.16, *p* = 0.004) and after the video (*ρ* = 0.14, *p* = 0.010). Regression analyses with robust (HC3) standard errors corroborated this finding (before: *β* = 0.18, *t*(315) = 2.91, *p* = 0.004; after: *β* = 0.16, *t*(315) = 2.74, *p* = 0.007). No significant association was observed between stress and change in PU (*ρ* = 0.01, *p* = 0.828).

**FIGURE 3:**
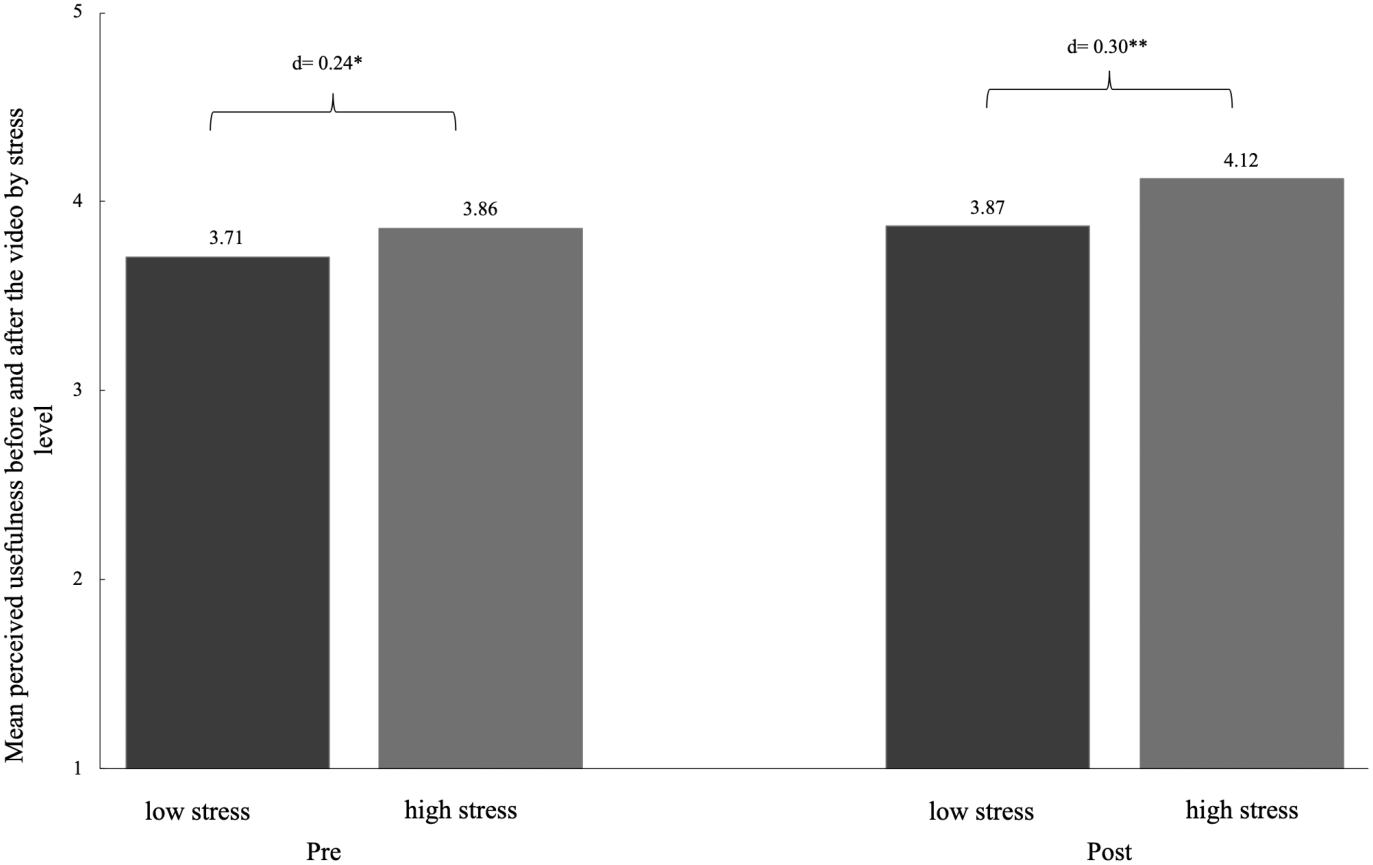
Mean perceived usefulness before and after the video by stress level. *N* = 317. Stress was dichotomised by median split for descriptive visualisation only. Primary analyses treated stress as a continuous variable. Between‐group comparisons are reported in Section 3. *p < 0.05; **p < 0.01; ***p < 0.001.

For descriptive purposes, participants with higher stress reported greater PU both before (*M* = 3.87 vs. 3.71; *t*(314.05) = 2.12, *p* = 0.035; *d* = 0.24) and after the stimulus (*M* = 4.12 vs. 3.86; *t*(310.55) = 2.72, *p* = 0.007; *d* = 0.30; Figure 3). No moderating effects of technological affinity, professional group, or self‐efficacy were identified.

### 3.5 Data Protection Concerns (RQ2)

No significant associations were observed between data protection concerns and PU before (*ρ* = −0.084, *p* = 0.135), after (*ρ* = −0.072, *p* = 0.201), or for the change score (*ρ* = 0.007, *p* = 0.895). Regression and group comparisons corroborated these findings (all *p*s non‐significant; smallest *p* = 0.11). An exploratory subgroup analysis revealed a significant negative interaction between data protection concerns and group membership (students, *n* = 262, vs. practitioners [trainee and licensed psychotherapists, including three participants with unspecified training status who were excluded from Table 1], *n* = 55), affecting intentions to use after the video (*b* = −0.46, SE = 0.16, *t*(313) = −2.98, *p* = 0.003, 95% CI [−0.77, −0.16]) as well as the change score (Δ PU [post–pre]; *b* = −0.30, SE = 0.12, *t*(313) = −2.43, *p* = 0.016, 95% CI [−0.53, −0.06]). Apart from this interaction, no direct associations between data protection concerns and PU were observed.

### 3.6 Age (RQ3)

Older participants reported lower PU both before (*ρ* = −0.13, *p* = 0.023) and after the video (*ρ* = −0.14, *p* = 0.013). Linear regressions corroborated these associations (before: *B* = −0.02, SE = 0.004, *t*(315) = −5.70, *p* < 0.001; after: *B* = −0.03, SE = 0.005, *t*(315) = −6.76, *p* < 0.001). Age was not significantly associated with the change in PU (*ρ* = −0.06, *p* = 0.305), indicating that the magnitude of the pre–post increase did not differ systematically with age. These effect sizes are small and should be interpreted with caution; age and professional group are confounded in this sample, as licensed psychotherapists were on average older than students.

### 3.7 Qualitative Findings: Personal Barriers (RQ4)

Open‐ended responses identified several recurring themes. Data protection and security were the most frequently cited concerns, encompassing fears about sensitive patient data, potential misuse, unauthorised access and data loss. Technical reliability and usability were also central, including concerns about system crashes, poor integration and non‐intuitive interfaces. Some participants feared negative effects on the therapeutic relationship. Practical challenges included training time and daily operational demands. Professional concerns included fears of skill erosion and over‐reliance on automated suggestions. Some participants reported limited prior familiarity with digital assistance systems.

## 4 Discussion

### 4.1 Summary and Discussion of Findings

This study examined PU of digital assistance systems in psychotherapy across psychology students, trainee psychotherapists and licensed psychotherapists. The primary findings are (1) PU increased significantly following a brief informational video stimulus, though this effect was negligible in licensed psychotherapists; (2) work‐related stress was positively associated with PU, contrary to the a priori hypothesis; (3) age was negatively but weakly associated with PU and (4) technological affinity, prior knowledge, self‐efficacy and data protection concerns showed no significant direct associations with PU.

The significant pre‐post increase in PU (*d* = 0.29) is consistent with TAM‐based predictions that context‐rich information enhances PU (Venkatesh and Davis 2000). However, the subgroup pattern requires careful interpretation. The effect was considerably larger in trainee psychotherapists (*d* = 0.59) and psychology students (*d* = 0.31) and was negligible in licensed psychotherapists (*d* = 0.06). Licensed psychotherapists may bring stronger prior knowledge, more critical evaluative frameworks, or already‐formed professional attitudes that are less amenable to brief informational interventions. Ceiling effects cannot be excluded. This finding may have implications for implementation research: brief video demonstrations alone may be insufficient to influence PU in practising clinicians, and more intensive or participatory approaches may be required for this group. The negligible change in this small subgroup may therefore indicate that brief demonstrations alone are insufficient when professional routines and accountability structures are already established.

The positive association between stress and PU was unexpected. One interpretation is that individuals under higher occupational strain perceive digital assistance systems as potentially useful coping resources, consistent with emerging work on digital resilience and technostress coping (Tiebel and Ernst 2025). Higher stress may increase the perceived need for support without necessarily increasing actual willingness to adopt a system. Thus, stress may act differently on distinct acceptance‐related constructs: it may enhance PU by increasing perceived need, while still potentially reducing ease of use, implementation readiness, or sustained use. However, given the small effect size (*ρ* ≈ 0.14–0.16), the cross‐sectional design and the student‐dominated sample, this interpretation remains speculative. Reverse causation and third‐variable explanations cannot be excluded. This finding should not be used as a basis for practical recommendations until replicated in practising samples.

The absence of significant direct associations for technological affinity, self‐efficacy and data protection concerns in the quantitative analyses may partly reflect restricted range in the predominantly young, student‐based sample. Notably, the qualitative responses indicated that data protection concerns remained prominent for at least some participants, consistent with findings from practitioner samples (Koch et al. 2020). Beyond restricted range, this discrepancy between non‐significant quantitative associations and salient qualitative privacy concerns suggests that data protection concerns may not directly reduce PU but may instead operate as implementation barriers at a later decision stage.

Age emerged as a consistent but small negative predictor of PU (*ρ* ≈ −0.13/−0.14). Given the small effect sizes and the confounding between age and professional group in this sample, age should not be interpreted as an isolated individual determinant; it likely functions as a proxy for professional status, clinical responsibility, cohort‐related technology exposure and established work routines. Older professionals, including licensed psychotherapists, may benefit from tailored informational formats and participatory design; however, the data do not provide sufficient grounds for strong practical recommendations in this regard.

From an implementation perspective, the qualitative findings may be particularly informative. They indicate that future adoption is unlikely to depend on PU alone, but also on whether systems are experienced as legally secure, technically reliable, time‐saving, clinically transparent and compatible with therapeutic autonomy.

### 4.2 Strengths and Limitations

Strengths include the pre–post design, use of validated instruments, robust statistical methods and a sample size exceeding a priori thresholds. Several limitations constrain the scope of conclusions. First, the sample was predominantly psychology students (82.6%), severely limiting generalisation to practising psychotherapists. This sample nonetheless offers some perspective on early professional socialisation, as these students are a relevant future user group. The negligible video effect in licensed psychotherapists is based on only *n* = 37 participants and cannot support firm conclusions. Second, trainee and licensed psychotherapists were combined for inferential subgroup analyses (*n* = 52 total) due to inadequate statistical power for separate group testing; meaningful differences between these groups may have been obscured. Third, video viewing was voluntary; variable completion rates introduce a potential confound in the pre‐post PU comparison. Fourth, scale adaptations (6‐point to 5‐point format for ATI‐S) limit comparability with normative data. Fifth, the video depicted a specific system (iptas); observed PU changes cannot be generalised to other digital assistance systems. Sixth, the exploratory nature of the study and the absence of multiple testing correction require that all *p* values be interpreted with particular caution. Seventh, the qualitative analysis rested on responses to an optional item from a self‐selected subset, limiting representativeness. As the open‐ended responses were coded by a single analyst, the thematic interpretation may be subject to individual bias, and no intercoder reliability could be established.

### 4.3 Future Directions

Future research should prioritise studies with larger and professionally diverse samples, adequate representation of licensed psychotherapists and longitudinal designs to examine whether PU translates into actual use. The unexpected positive association between stress and PU warrants replication and mechanistic investigation. Intervention research should compare different information formats in building sustained PU, particularly among experienced clinicians. The present findings do not provide a sufficient empirical basis for strong recommendations regarding training programmes for older professionals; such recommendations require further empirical support.

## 5 Conclusion

This study provides preliminary evidence that a brief video demonstration may increase PU of a digital assistance system, primarily among psychology students and trainee psychotherapists. Effects were negligible in licensed psychotherapists, indicating that a brief informational stimulus appeared insufficient in this small subgroup. Work‐related stress showed an unexpected positive association with PU that requires replication before conclusions can be drawn. Age was consistently but weakly negatively associated with PU. The findings suggest that future studies and implementation efforts should differentiate between professional groups. Rigorous longitudinal and practice‐based studies are needed to assess the potential of digital assistance systems in psychotherapy.

## Author Contributions

**Michelle Oberthaler:** conceptualization, writing – original draft, data curation, formal analysis, writing – review and editing, methodology, project administration. **Bonnie Röhrig:** conceptualization, project administration, writing – review and editing, validation. **Frank Petrak:** methodology, writing – review and editing, supervision, validation, conceptualization, investigation. **Stephan Goerigk:** validation, data curation, writing – review and editing, methodology.

## Funding

The authors have nothing to report.

## Ethics Statement

This study was conducted in accordance with the ethical principles of the 1964 Declaration of Helsinki and its later amendments. As an anonymous, minimal‐risk online survey including an informational video stimulus, with no clinical intervention and no collection of sensitive personal data, the study did not require formal ethics committee approval under applicable German regulations. All participants provided informed electronic consent prior to participation.

## Consent

Informed consent was obtained from all participants prior to their participation.

## Conflicts of Interest

The authors declare no conflicts of interest. Frank Petrak reports a potential conflict of interest due to his role as founder and managing director of iptas INNOVATIONS GmbH, which develops digital assistance systems for psychotherapy. He is also affiliated with the LWL University Hospital for Psychosomatic Medicine and Psychotherapy, Ruhr University Bochum, where he conducts research.

## Data Availability

The data that support the findings of this study are available from the corresponding author upon reasonable request.
